# Factors influencing treatment outcomes assessed by the American Board of Orthodontics Objective Grading System (ABO-OGS)

**DOI:** 10.1186/s12903-023-03735-z

**Published:** 2023-12-14

**Authors:** Tanyapak Kongboonvijit, Sirichom Satrawaha, Anupap Somboonsavatdee

**Affiliations:** 1 Department of Orthodontics, Faculty of Dentistry, Chulalongkorn University, 34 Henri-Dunant Road, Wangmai, Pathumwan, Bangkok, 10330 Thailand; 2 Department of Statistics, Chulalongkorn Business School, Chulalongkorn University, Bangkok, 10330 Thailand

**Keywords:** American Board of Orthodontics Objective Grading System index, Discrepancy index, Treatment outcomes, Camouflage, Orthognathic surgery

## Abstract

**Background:**

Treatment outcomes can be influenced by various factors. This study aimed to determine the association between predisposing patient- and treatment-related factors (demographic, cephalometric parameters, skeletal relationships, Discrepancy Index (DI), extractions, treatment type and duration) and treatment outcomes measures according to the American Board of Orthodontics Objective Grading System index (ABO-OGS).

**Methods:**

Completed cases (*N* = 100) were included in this cross-sectional study. One calibrated examiner assessed DI, pretreatment lateral cephalometric parameters and ABO-OGS. Patient data, including sex, age, types of malocclusion, extractions, treatment type, and duration, were also collected. Intraexaminer reliability for each measurement was evaluated using the intraclass correlation coefficients. Multiple linear regression analysis, using the backward elimination method with a significance level (α) of 0.05, was used to determine which factors significantly influenced the ABO-OGS score.

**Results:**

From the study, the overall mean ABO-OGS score was 11.36 points. Factors influencing the ABO-OGS score were pretreatment Wits values (*p* value = .000), L1-NB (°) (*p* value = .023) and treatment duration (*p* value = .019). Subjects with lower negative values of Wits and L1-NB (°) tended to have higher ABO-OGS scores. Additionally, the ABO-OGS score tended to be higher for subjects with longer treatment times.

**Conclusions:**

The majority of treated subjects had satisfactory orthodontic treatment outcomes assessed by the ABO-OGS. The pretreatment severity of skeletal discrepancies determined by the Wits parameter, the degree of retroclined lower incisors and longer treatment duration negatively impacted the treatment outcomes.

**Supplementary Information:**

The online version contains supplementary material available at 10.1186/s12903-023-03735-z.

## Background

In orthodontics, the clinician is confronted with many different types of tooth and jaw misalignment that can each be treated in different ways to achieve the desired treatment outcomes. However, each clinician implements the concepts of orthodontic treatment based on their own preferences and experience. To guarantee the quality of orthodontic management and establish an ideal standard treatment, the utilization of quantitative outcome assessments should be prioritized. The American Board of Orthodontics Objective Grading System (ABO-OGS) is a widely accepted and extensively reported index that was introduced by the ABO in 1998 to meet the highest standards of orthodontic treatment outcomes. Dental casts and panoramic radiographs, which are standard clinical records taken for orthodontic diagnosis and treatment planning, were also used to assess this index [1].

Higher ABO-OGS scores indicate more discrepancy from the ideal treatment outcomes. An ideal score was 0. Cases that scored 27 points or fewer were in the pass group. The measurement criteria are alignment, marginal ridges, buccolingual inclination, occlusal contacts, occlusal relationship, overjet, interproximal contacts and root angulation [1]. Based on the correlation coefficient values (*r*) for intraexaminer and interexaminer reliability, the ABO-OGS showed a good reliable and reproducible index [2]. Consequently, many studies have used the ABO-OGS assessment to evaluate orthodontic clinical outcomes [3–7].

The ABO was also developed using the Discrepancy Index (DI) to evaluate the pretreatment severity of a patient’s malocclusion. The measurements of overjet, overbite, anterior open bite, lateral open bite, crowding, occlusion, lingual posterior crossbite, buccal posterior crossbite, ANB angle, IMPA and SN-GoGn angle were brought up to the total DI scores. A higher DI score indicates a more complex case [8].

Treatment outcomes can be affected by various factors, including patient-, operator- and appliance-related factors [9]. However, whether these factors can truly predict treatment outcomes remains a controversial issue [3, 5–7, 10–15]. For the types and complexity of malocclusion assessed by the DI, Class I malocclusion had a higher odds ratio for obtaining a passing ABO-OGS score compared to other malocclusions [11]. The more complex cases were correlated with higher ABO-OGS scores [5]. The duration of treatments also predicted clinical occlusal outcomes. The longer treatment period was correlated with the poorer quality of the outcomes [13, 14]. Meanwhile, for patient age, sex and types of orthodontic treatment (early- vs late-treatment groups), there was no correlation to the final quality assessed by the ABO-OGS score [10, 12]. To date, no study has evaluated the association between all these potential predictors, encompassing cephalometric parameters, and clinical occlusal outcomes.

This study’s objective was to determine the association between patient-related (sex, age at start, types and complexity of malocclusion, cephalometric parameters) and treatment-related predisposing factors (extractions, treatment type, and treatment duration) and treatment outcomes measures according to the ABO-OGS index.

## Methods

### Study design and setting

This cross-sectional study was retrospectively conducted from the finished cases between January 2017 and December 2021. The cases had been treated by several orthodontic residents at the postgraduate orthodontic clinic, Faculty of Dentistry, Chulalongkorn University. Ethical approval for the study was given by the Human Research Ethics Committee (HREC-DCU 2020–115 approved on 4/12/2020). All markings that could identify the subjects and the clinicians were removed from all the records.

### Inclusion and exclusion criteria

The criteria for the inclusion of subjects were (1) Thai ethnicity only, (2) full-mouth edgewise appliances, (3) permanent dentition and (4) completed treatment records, which included dental casts, lateral cephalograms and panoramic radiographs. Subjects with craniofacial syndromes and/or early bracket debonding were excluded from this study.

### Sample size calculation

G*Power software version 3.1 was utilized to perform statistical power analyses for the calculation of the sample size in this study [16]. In accordance with Cohen’s criteria, a medium effect size (*f*^2^ = 0.15) and eighteen predictors were considered. The outcome of this analysis determined that a total of 90 subjects was required for the sample size. The sample selection was conducted through a multistage stratified random sampling method. In this multistage stratified random sampling process, the initial segmentation of the population was based on the types of malocclusion. Subsequently, further stratification was carried out with regard to gender, age, and types of treatment respectively. In total, 100 participants who met the inclusion and exclusion criteria were included in this study.

### Independent variables

Patient factors included age, sex, types of malocclusion (Class I, II, and III assessed by ANB and Angle’s classification), pretreatment discrepancy index (DI) and pretreatment lateral cephalometric parameters. The DI uses 11 criteria, including overjet, overbite, anterior open bite, lateral open bite, crowding, occlusion, lingual posterior crossbite, buccal posterior crossbite, ANB, IMPA and SN-GoGn angle. In the current application of lateral cephalometric analysis, all radiographs were digitally traced and analyzed according to the ABO [17]. For treatment factors, extractions, treatment duration and types of treatment were assessed. Types of treatment were categorized into three groups, comprising baseline orthodontic treatment, camouflage treatment, and orthognathic surgery. The baseline orthodontic treatment group represents patients with normal skeletal relationships who are undergoing fixed orthodontic treatment. In contrast, individuals with skeletal discrepancies have been categorized into either the camouflage or orthognathic surgery group.

### Dependent variables

Treatment outcomes were assessed by the ABO-OGS index [1]. To determine this index, all casts and panoramic radiographs were measured. The scores for each of the criteria were added together to assess for any discrepancy from the ideal occlusion. All variables under investigation are displayed in Table 1.

**Table 1:** Variable data

Patient factors	Treatment factors	Treatment outcome assessment
- Sex - Age - Types of malocclusion (Class I, II or III) - DI score - Pretreatment cephalometric parameters (ANB, FMA, Wits, U1-NA (°), U1-NA (mm), L1-NB (°), L1-NB (mm), upper lip to E-line (mm), lower lip to E-line (mm), nasolabial angle (NLA) and H-angle)	-Extractions -Types of treatment (Baseline orthodontic treatment, Camouflage treatment, Orthognathic surgery) -Treatment duration (months)	- ABO-OGS score

### Examiner calibration and reliability

Multistage stratified random sampling was used to reduce the risk of selection bias. Before the commencement of the study, the examiner was calibrated to an American board-certified specialist to assess the interexaminer reliability by measuring DI, lateral cephalometric values and the ABO-OGS index for 20 randomly selected cases. In addition, to evaluate intraexaminer reliability, one researcher also measured those parameters for 20 randomly chosen subjects two times within a 1-week interval. Patient and treatment factors were unaffected and free from bias, as this information was collected after the scoring index had been undertaken. Intraexaminer and interexaminer reliability analyses were determined by calculating intraclass correlation coefficients (ICCs).

### Statistical analysis

All data were imported from Excel into IBM SPSS software version 22.0 for Windows (IBM Corp., Armonk, NY, USA), and all statistical analyses were conducted using this application. A normality test was performed and showed normally distributed residuals from the analysis. Collinearity statistics were employed to examine potential multicollinearity among the predictors, ensuring the absence of confounding variables. Multiple linear regression analysis using the backward elimination method with a significance level (α) of 0.05 was employed to identify influencing factors for the ABO-OGS total score. The models examined the primary outcome, the ABO-OGS total score, in relation to demographic factors, potential predictors, and confounding variables such as sex, age, types of malocclusion, DI score, pretreatment lateral cephalometric parameters, extractions, types of treatment, and treatment duration. Spearman’s rank correlation was used to evaluate the strength of the association between independent variables and the ABO-OGS total score. The level of statistical significance was set at 0.05.

## Results

### Demographic data

The demographic data of all subjects (*N* = 100) were collected (Tables 2 and 3). The mean age of the entire sample was 19.46 years, ranging from age 12 to 48 years. Of all subjects, 59% were aged < 20 years, and 41% were aged > 20 years. The male to female ratio was 21:29. Thirty-three percent had Class I malocclusion, 33% had Class II malocclusion, and 34% had Class III malocclusion. The average DI score (pretreatment complexity) for the entire sample was 25.74 points. Fifty-nine percent had extraction therapy. In addition, 48% received camouflage treatment (Class II 62.5% and Class III 37.5%), 20% received orthognathic surgery (Class I 5% Class II 15% and Class III 80%), and 32% were categorized in baseline orthodontic treatment. The average treatment duration was 36.18 months, ranging from 14 to 57 months (Tables 2 and 3).

**Table 2:** Distribution of demographic and patient-related variables

Variables			Frequency (%)	
**Sex**	Male		42	
	Female		58	
**Age**	< 20 years old		59	
	≥20 years old		41	
**Types of malocclusion**	Class I		33	
	Class II		33	
	Class III		34	
Variables		Mean (SD)	Min	Max
**DI score**	total	25.74 (1.61)	6	92
	Class I	15.12 (7.05)	6	30
	Class II	30.09 (12.93)	13	87
	Class III	31.82 (19.93)	6	92
**Pretreatment cephalometric parameters** (Norm Mean ± SD)	SNA (85 ± 4)	83.47 (3.47)	76.00	93.00
	SNB (82 ± 3)	81.00 (4.97)	70.50	93.00
	ANB (3 ± 2)	2.53 (4.18)	−14.00	12.00
	Wits (−3 ± 2)	−2.60 (6.20)	−19.50	9.50
	FMA (25 ± 4)	23.81 (6.74)	8.00	46.00
	U1-NA (°) (28 ± 4)	28.46 (9.19)	5.00	52.00
	U1-NA (mm) (6 ± 2)	7.39 (4.14)	−5.00	28.00
	L1-NB (°) (32 ± 6)	29.64 (6.78)	13.00	42.00
	L1-NB (mm) (6 ± 2)	7.51 (2.68)	0.00	14.00
	U1-L1 (118 ± 8)	119.94 (14.07)	87.50	163.00
	Upper lip to E-line (−1 ± 2)	0.98 (3.06)	−7.00	8.50
	Lower lip to E-line (2 ± 2)	3.22 (2.73)	−3.00	10.50
	NLA (89 ± 11)	87.86 (10.06)	56.50	108.00
	H-angle (14 ± 4)	15.27 (5.69)	−4.00	28.00

**Table 3:** Distribution of treatment-related variables

Variables		Frequency (%)		
**Extractions**	Nonextraction	41		
	2 premolars	17		
	3 premolars	7		
	4 premolars	29		
	Other (incisors and molars)	6		
**Types of treatment**	Baseline treatment	32		
	Camouflage	48		
	Orthognathic surgery	20		
**Variables**		**Mean (SD)**	**Min**	**Max**
**Treatment duration**		36.18 (8.36)	14	57

### Treatment outcomes

The ICC values for interexaminer and intraexaminer reliability ranged from 0.889 to 0.986 and 0.973 to 0.989, respectively which indicating good reliability [18]. The mean ABO-OGS score was 11.36 points. The majority of subjects were classified as passing the ABO-OGS score (98%), while two subjects with Class III malocclusion, one of whom was treated with orthognathic surgery and the other subject was treated with camouflage treatment, were categorized in the failure group (Table 4).

**Table 4:** Distribution of the ABO-OGS scores (main treatment outcome)

ABO-OGS	Frequency (%)	Mean (SD)	Min	Max
Total	100	11.36 (6.35)	2	29
Passed group	98	11 (5.88)	2	27
Failed group	2	29	29	29
Class I	33	10.03 (5.50)	3	25
Class II	33	8.58 (4.11)	3	14
Class III	34	15.35 (7.02)	5	29

### Predictors of treatment outcomes

**Fig. 1:**
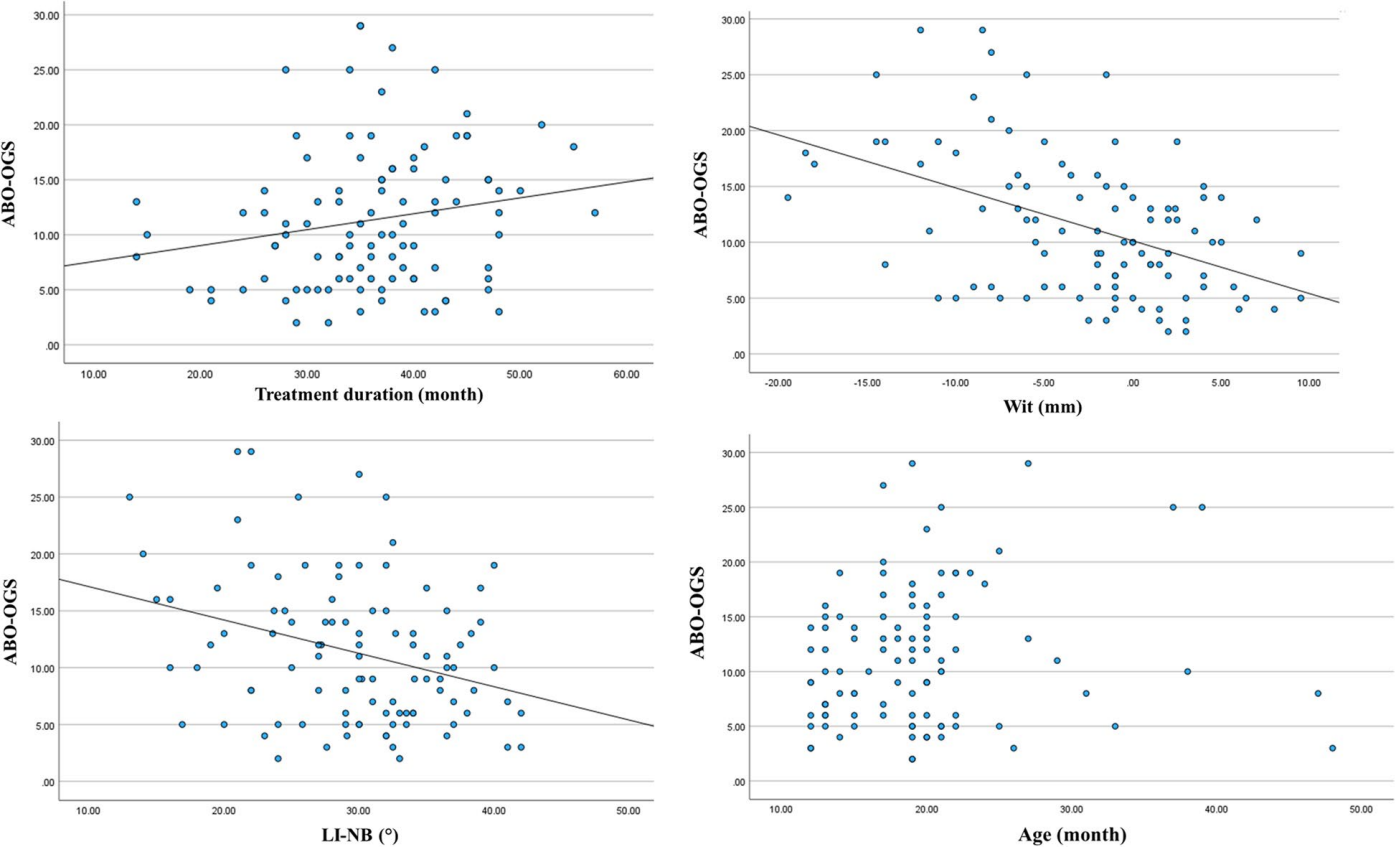
The scatter plot between factors, treatment duration, Wit, LI-NB (°), age, and ABO-OGS

To determine the potential predictors (sex, age, types of malocclusion, DI score, pretreatment lateral cephalometric parameters, extractions, types of treatment and treatment duration), multiple linear regression analysis was conducted using the backward elimination method. The results in Table 5 show that Wits (*p* value = .000), L1-NB (°) (*p* value = .023) and treatment duration (*p* value = .019) were the factors influencing the ABO-OGS score. The multiple linear regression analysis model was statistically significant in predicting the ABO-OGS score (*p* value <.001, R^2^ = .317) ABO-OGS score = 8.856+ (0.517xduration) – (0.377xWit) – (0.199x L1-NB (°)). The regression coefficients indicated that an increased treatment duration, lower negative pretreatment Wit and LI-NB (°) by 1 unit (month, mm and degree, respectively) are associated with a change in ABO-OGS score of .517, −.377 and − .199, respectively (dependent on all other variables being constant) (Table 5). Scatter plots with trend lines showed a significant Spearman positive correlation between Duration versus ABO-OGS (r = .211; *p* value = .035), a negative correlation between Wit versus ABO-OGS (r = −.424; *p* value <.001), a negative correlation between LI-NB (°) versus ABO-OGS (r = −.273.; *p* value = .006), and no significant correlation between Age versus ABO-OGS (Fig. 1).

**Table 5:** Multiple linear regression of variable significance testing against outcome: ABO-OGS total score

Variables		β (SE.)	*p* value
**Patient factors**	Age	.157(.066)	.078
	Wits	−.377(.093)	.000*
	L1-NB (°)	−.199(.086)	.023*
**Treatment factors**	Treatment duration	.157 (.060)	.019*

β regression coefficient, SE standard error, *p value< 0.05 was considered statistically significant

## Discussion

The objective of this study was to investigate the factors influencing the ABO-OGS score. Our multiple linear regression analysis revealed that the ABO-OGS score can be predicted based on a given independent variable while controlling for all other variables, resulting in an R-squared value of 31.7%. The Wits values are the most influential factor on the ABO-OGS score, with lower negative values of the Wits cephalometric parameter predicting a higher ABO-OGS score. Additionally, we performed Spearman’s rank correlation to investigate the strength of the correlation. The results showed a significant negative correlation between the Wits values and the ABO-OGS total score. In our Class III malocclusion subjects, the Wits values ranged from − 3 to − 19.5. This may indicate the possibility of poorer outcomes in subjects with dental base class III. This finding implies the significance of the Wits values in predicting orthodontic treatment outcomes. Although our study did not demonstrate a significant influence of the type of malocclusion, as evaluated by ANB and angle classification, on ABO-OGS scores, it is worth noting that Class III malocclusion exhibited the highest discrepancy index (DI). Consequently, treatment for Class III malocclusion tends to be more challenging, complex, and difficult to manage, resulting in higher ABO-OGS scores. Specifically, Class III malocclusion displayed the highest mean ABO-OGS score (Tables 2 and 4). These findings have not been previously documented in the literature; nevertheless, they are consistent with our hypotheses. The primary issue with the ANB angle is its susceptibility to individual variations in craniofacial physiognomy due to the usage of cranial reference planes. There were some shortcomings of the ANB angle compared to Wit values, including position of N, and vertical features of the samples [19–23]. Thus, the ANB angle does not always indicate the reliable severity of malocclusion. These factors can cause differences in the influence of these ANB and Wit values on ABO-OGS scores. On the other hand, studies by Struble and Huang [11] found that subjects with Class I malocclusion as assessed by Angle’s classification were more likely to achieve higher percentages of passing ABO-OGS scores compared to other types of malocclusion. In our study, we categorized the type of malocclusion using both Angle’s classification and the ANB angle to confirm the skeletal relationship and minimize errors arising from variations in the first molar position, such as early loss of primary teeth, mesial drift, and tooth tipping or crowding. These factors may contribute to differences in the impact of malocclusion type on treatment outcomes.

There was also a negative coefficient for L1-NB (°), which indicated that subjects with retroclined lower incisors, which are also characteristic of Class III malocclusion, tended to have higher ABO-OGS scores and poorer treatment outcomes. Meanwhile, the higher of these values, normal inclination or proclination of lower incisors, which were the characteristic of Class I and II malocclusion related to the lower ABO-OGS score, better treatment outcomes. A significant positive correlation was found between L1-NB (°) and Wit (*p* value = .001), with a correlation coefficient of r = .323, suggesting a consistent relationship between these two factors (data not shown). Subjects with positive Wit values tend to have more proclined lower incisors. This characteristic is often observed in Class II malocclusion, which is associated with lower ABO-OGS scores.

For other patient factors, including sex, age, DI score and all pretreatment lateral cephalometric values except Wits and L1-NB (°), there was also no significant association detected within the regression analysis in ABO-OGS outcome. Previous study [10] found no influence of sex, age at start, pretreatment ANB or the mandibular plane angle on treatment outcomes. Meanwhile, Birkelain et al. [24] stated that age at start accounted significantly for the amount of improvement in outcome quality assessed by the Peer Assessment Rating index (PAR). Some adults have a limited amount of tooth movement due to periodontal conditions, or their orthodontic treatment only aims for preprosthetic correction; thus, they received the lesser quality of treatment outcomes. For the pretreatment DI score, we found no association with the final ABO-OGS score. This concurs with some previous studies that also found no correlation between these two indices [3, 7]. Campbell et al. [5] found a significant correlation between these two scores. They indicated that for every 1 point increase in the DI, the ABO-OGS increased by 0.23 ± 0.06 points. Likewise, Pulfer et al. [15] reported weak positive relationships of DI score to ABO-OGS score.

This study also determined what treatment-related factors (extractions, treatment type and duration) can predict the ABO-OGS score. Only treatment duration was a factor that influenced the ABO-OGS score. The average treatment period in our study was 36.18 ± 8.36 months, slightly longer than the durations reported in other graduate orthodontic program studies, which were 25 and 33.94 months [13, 24]. The complexity of cases treated within our university program, particularly surgical cases, contributed to the extended total treatment times. The coefficient of the factor was positive (β = .517), indicating that subjects with longer treatment times had higher ABO-OGS scores. Furthermore, an association was found between pretreatment Wits values and treatment duration with a negative coefficient (β = −.650; *p* value = .073; r^2^ = .237) (data not shown).) The results indicated that patients with lower negative Wits values, indicating greater severity of dental base Class III, were more likely to receive longer treatment times, resulting in higher ABO-OGS scores. These findings are supported by Pinskaya et al. [13], who showed progressively lessened treatment outcomes of completed cases that were associated with a treatment time increase from 28.9 to 39.3 months. More appointments and extended treatment time might have caused the declining patient compliance in cases of poorer oral hygiene and mechanics. Fink and Smith [25] also reported a positive correlation coefficient between treatment duration and the number of missed appointments. In contrast, some studies [26, 27] found no connection between the length of the treatment and the occlusal outcomes. Sunanta et al. [7] also found no significant difference in total treatment duration among ABO-OGS groups. For extraction consideration and treatment type, we found no significant influence on the quality of treatment outcomes. Although the patients who underwent extraction (37.14 ± 7.19) required a longer treatment time than nonextraction patients (34.80 ± 9.72), there was no significant difference between these two groups. In contrast, Pinskaya et al. [13] found a statistically significant difference between the treatment duration of extraction versus nonextraction patients. Papageorgiou et al. [27] also found that comprehensive treatment involving extraction of the four premolars may lead to potentially better occlusal outcomes.

### Limitations and recommendations

The limitation of this retrospective study is that the samples were relatively homogenous from a graduate orthodontics program, which cannot be applied to all orthodontics treatment outcome quality. Since there are many factors influencing treatment outcomes, no single indicator or measure is now able to capture them adequately. Moreover, other appropriate contributing factors, including different mechanics or treatment modalities, require further investigation. The experience and skill of orthodontists and patient cooperation, which is difficult to control and record, might also affect treatment outcomes [28]. Furthermore, evaluation of long-term outcomes in the retention period should also be considered.

### Clinical applications

The ABO-OGS index is a measurably and objectively assessment for evaluating orthodontic treatment outcomes, which is valuable for educational objectives. The evaluation helps to improve, provide an ideal standard and guarantee the quality of treatment outcomes. Considering predisposing factors that influence the orthodontic treatment outcomes helps the clinician to consider cases that are prone to poorer results, which are subjects with lower Wits and L1-NB (°) values. A longer treatment duration can also result in a lower quality of treatment outcomes. Prolonged treatment time will lead to a lack of patient cooperation in keeping appointments, poor hygiene compliance, and disregard for biomechanical instructions.

The appropriate duration of treatment, contingent upon the severity of the malocclusion, generally leads to greater benefits for patients.

## Conclusions

Overall, according to the ABO-OGS score, the majority of cases completed from the graduate orthodontic clinic were in the pass group. Subjects with initial lower negative values of Wits and L1-NB (°) produced poorer outcomes. Meanwhile, subjects with shorter treatment durations obtained higher orthodontic occlusal outcomes based on the ABO-OGS total score.

### Supplementary Information

**Additional file 1: Table 1. **The Spearman’s rank correlation coefficients (*r*) between L1-NB (°), ANB and Wit. **Table 2.** The multiple linear regression analysis of variables testing against treatment duration

## Data Availability

The datasets used and/or analyzed during this study belong to the authors and are available from the corresponding author only upon reasonable request.

## Abbreviations

ABO-OGS: American Board of Orthodontics Objective Grading System index
DI: Discrepancy Index
PAR: Peer Assessment Rating
ANB: Point A-Nasion-point B
Wits appraisal: AB/Occlusal plane angle
ICC: Intraclass Correlation Coefficients
