# Supplementary material for: Factors influencing treatment outcomes assessed by the American Board of Orthodontics Objective Grading System (ABO-OGS)
Source: BMC Oral Health. 2023 Dec 14;23:1000. doi: 10.1186/s12903-023-03735-z (PMC10720172; doi:10.1186/s12903-023-03735-z)
Supplement: Supplementary file 1 — Additional file 1: Table 1. The Spearman’s rank correlation coefficients (r) between L1-NB (°), ANB and Wit. Table 2. The multiple linear regression analysis of variables testing against treatment duration [file 12903_2023_3735_MOESM1_ESM.docx]

**Supplementary Data**

Table 1. The Spearman’s rank correlation coefficients (*r*) between L1-NB (°), ANB and Wit

| **Variables** | **Wit** | |
| --- | --- | --- |
|  | ***r*** | ***p* value** |
| **L1-NB (°)**  **ANB** | .323  .871 | .001*  <.001* |

r correlation coefficient, *p value<0.05 was considered statistically significant.

Table 2. The multiple linear regression analysis of variables testing against treatment duration

| **Variables** | | **β (SE.)** | ***p* value** |
| --- | --- | --- | --- |
| **Patient factors** | Sex  Age  Types of malocclusion  -Class I  -Class II  -Class III  DI score  ANB  Wits  FMA  U1-NA (°)  U1-NA (mm)  L1-NB (°)  L1-NB (mm)  Upper lip to E-line  Lower lip to E-line  NLA  H-angle | -.207(1.969)  -.125(.138)  -1.484(6.886)  3.814(4.999)  0^a^  .021(.073)  -.296(.733)  -.650(.358)  -.200(.157)  .107(.176)  -.551(.380)  -.057(.262)  1.170(.732)  1.202(.710)  -1.434(.541)  .059(.095)  -.095(262) | .917  .371  .769  .448  -  .769  .688  .073  .205  .545  .151  .829  .114  .095  .010  .538  .719 |
| **Treatment factors** | Extractions  Types of treatment  -Baseline orthodontic treatment  -Camouflage treatment  -Orthognathic surgery | -2.449(2.070)  .004(6.644)  -1.320(6.702)  0^a^ | .240  .999  .844  - |

β regression coefficient, SE standard error, a The parameter is set to zero, R Squared = .237.
